# Assessment of Water Quality in a Subtropical Alpine Lake Using Multivariate Statistical Techniques and Geostatistical Mapping: A Case Study

**DOI:** 10.3390/ijerph8041126

**Published:** 2011-04-15

**Authors:** Wen-Cheng Liu, Hwa-Lung Yu, Chung-En Chung

**Affiliations:** 1 Department of Civil Disaster Prevention Engineering, National United University, Miao-Li, 36003, Taiwan; E-Mail: w933821@hotmail.com; 2 Department of Bioenvironmental Systems Engineering, National Taiwan University, Taipei, 10617, Taiwan; E-Mail: hlyu@ntu.edu.tw

**Keywords:** multivariate statistical technique, geostatistical mapping, water quality, principal component analysis, cluster analysis, Yuan-Yang Lake

## Abstract

Concerns about the water quality in Yuan-Yang Lake (YYL), a shallow, subtropical alpine lake located in north-central Taiwan, has been rapidly increasing recently due to the natural and anthropogenic pollution. In order to understand the underlying physical and chemical processes as well as their associated spatial distribution in YYL, this study analyzes fourteen physico-chemical water quality parameters recorded at the eight sampling stations during 2008–2010 by using multivariate statistical techniques and a geostatistical method. Hierarchical clustering analysis (CA) is first applied to distinguish the three general water quality patterns among the stations, followed by the use of principle component analysis (PCA) and factor analysis (FA) to extract and recognize the major underlying factors contributing to the variations among the water quality measures. The spatial distribution of the identified major contributing factors is obtained by using a kriging method. Results show that four principal components *i.e.*, nitrogen nutrients, meteorological factor, turbidity and nitrate factors, account for 65.52% of the total variance among the water quality parameters. The spatial distribution of principal components further confirms that nitrogen sources constitute an important pollutant contribution in the YYL.

## 1. Introduction

Water quality is the main factor controlling healthly and diseased states in both humans and animals. Surface water quality is an essential component of the natural environment and a matter of serious concern today. The variations of water quality are essentially the combination of both anthropogenic and natural contributions. In general, the anthropogenic discharges constitute a constant source of pollution, whereas surface runoff is a seasonal phenomenon which is affected by climate within the water catchment basin [1]. Among them, because of the intensive human activities, the anthropogenic inputs from a variety of sources are commonly the primary factors affecting the water quality of most rivers, lakes, estuaries, and seas, especially for those close to highly urbanized regions.

Many investigations have been conducted on anthropogenic contaminants of ecosystems [2–4]. Because of the spatial and temporal variations in water quality conditions, a monitoring program which provides a representative and reliable estimation of the quality of surface waters is necessary. The monitoring results produce a large and complicated data matrix that is difficult to interpret to draw meaningful conclusions. Multivariate statistical techniques are powerful tools for analyzing large numbers of samples collected in surveys, classifying assemblages and assessing human impacts on water quality and ecosystem conditions.

The application of different multivariate statistical techniques, such as principal component analysis (PCA), factor analysis (FA), cluster analysis (CA), and discriminate analysis (DA), assists in the interpretation of complex data matrices for a better understanding of water quality and ecological characteristics of a study area. These techniques provide the identification of possible sources that affect water environmental systems and offer a valuable tool for reliable management of water resources as well as rapid solution for pollution issues [5–7]. Multivariate statistical techniques have been widely adopted to analyze and evaluate surface and freshwater water quality, and are useful to verify temporal and spatial variations caused by natural and anthropogenic factors linked to seasonality [8,9].

Geostatistical mapping is based on field observations. Because field surveys are limited by the cost of sampling, only sparse observation data are generally available. Geostatistical mapping or further analysis requires the assessment of exhaustive attribution values for an entire study area. Geostatistical mapping techniques have been widely applied to different fields including water quality in bays [10] watersheds [11], soil properties [12], precipitation [13], river discharges [14], air pollution [15], and so on. To the best of our knowledge, geostatistical mapping has not been adopted for studying water quality data in lakes.

The objective of the present study was to analyze 14 physico-chemical water quality parameters in water samples collected on monthly basis from 2008 to 2010 in a subtropical alpine lake (Yuan-Yang Lake) in Taiwan. The data matrix obtained from field measurement was subjected to the CA, PCA, and FA techniques, as well as geostatistical mapping to evaluate information about the similarities between sampling stations and to ascertain the important contributions of nutrient sources among water quality parameters in the alpine lake.

## 2. Materials and Methods

### 2.1. Study Site and Sample Collection

**Figure 1.**
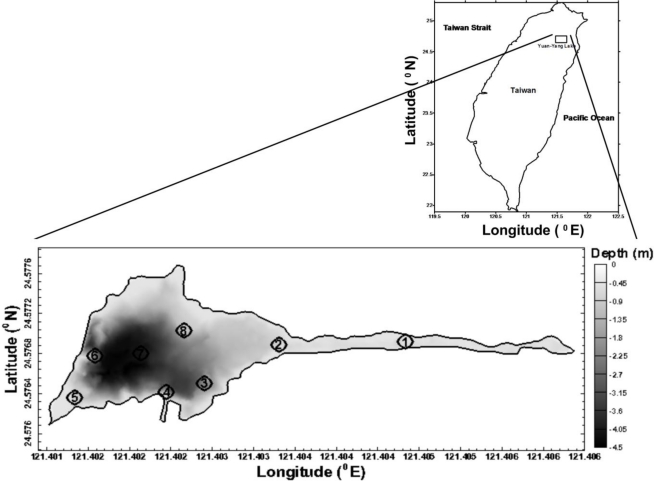
Location of Yuan-Yang Lake (YYL) in Taiwan and eight measurement stations in YYL.

The Long-Term Ecological Research (LTER) program is one of the core projects of the Global Change and Terrestrial Ecosystem program (GCTE), which is under the umbrella of the International Geosphere-Biosphere Program (IGBP). An understanding of ecological processes and of mechanisms leading to ecologically tragic events is particularly important for the sustainability of Taiwan Island. To meet such a requirement, the LTER project was initiated in 1992 on the island. Yuan-Yang Lake (YYL) is one of the six LTER sites and the only site associated with a mountain lake ecosystem in Taiwan. YYL, a small (3.6 ha) and shallow (4.5 m maximum depth) lake in a mountainous catchment 1,730 m above sea level, is located in the northeastern region of Taiwan (24°35′ N, 121°24′E) (Figure 1). The lake and surrounding catchment (374 ha) were designated as a long-term ecological study site by the Taiwan National Science Council in 1992 and joined the Global Lake Ecological Observatory Network (GLEON) in 2004. The lake is an important site for studying physical characteristics, water quality, and ecosystems. Recently, the lake has been subject to pollution sources from recreational activities, therefore the investigation of water quality is urgent and necessary.

The steep watersheds are dominated by pristine Taiwan false cypress [*Chamaecyparis obtusa* Sieb. & Zucc. var. formosana (Hayata) Rehder] forest. The average annual temperature is approximately 13 °C (monthly average ranges from −5 to 15 °C) and the annual precipitation is more than 4,000 mm. YYL is subject to three to seven typhoons in summer and autumn each year, during which more than 1,700 mm of precipitation may fall on the lake.

The sampling network including eight measured stations was designed to cover a wide range of key locations accounting for inflow and outflow (Figure 1). Stations 1 and 2 are located at shallow area which is a swamp (shallow) zone. Stations 3 to 8 are located at the middle and deep zones. Station 4 is near by water inflow site, while station 5 is close to the site of lake water outflow.

Water temperatures were measured through the water column at 0.5 m increments using a thermistor chain (Templine, Apprise Technologies, Inc. Duluth, MN, USA). Wind speed was measured 1 m above the lake by an anemometer (model 03001, R.M. Young, Traverse, MI, USA). Precipitation, air temperature and downwelling photosynthetically active radiation (PAR) were measured at a land-based meteorological station approximately 1 km away from the lake. Variation in water levels was measured using a submersible pressure transmitter [PS 9800(1), Instrumentation Northwest, Kirkland, WA, USA] deployed at the lake shore (Figure 1). The attenuation of irradiance by the water column, in the 400–700 nm bands, was measured using a Licor underwater quantum flat head sensor. The outputs from the senor were stored using Licor data logger in the field, and converted to light measurements in the laboratory.

The pH, turbidity, and Secchi depth were measured *in situ*. Dissolved oxygen concentration was measured with a dissolved oxygen meter (Yellow Springs Instruments Company USA, Model 550A). The water samples, collected using an open water grab sampler equipped with a sample pull-ring that allowed for sampling at different water depths, were analyzed and measured in laboratory to obtain total suspended solids (TSS), nutrients (nitrate nitrogen, ammonium nitrogen, total nitrogen, and total phosphorus), and chlorophyll a concentrations. Chlorophyll a was measured by filtering with 600 cm^3^ samples through a glass fiber filter. The filter paper itself was used for the analysis. The filtering was group up 90% acetone solution and fluorometer is used to read the light transmission, which in turn was used to calculate the concentration of chlorophyll a. TSS and nutrients, concentration was analyzed using the US EPA standard method 160.1 [16].

### 2.2. Cluster Analysis

CA is an unsupervised pattern recognition method that divides a large amount of cases into smaller groups or clusters based on the characteristics they process. The resulting clusters of objects should exhibit high internal (within cluster) homogeneity and high external (between clusters) heterogeneity. Hierarchical CA is the most common approach, which starts with each case in a separate cluster and joints the clusters together step by step until only one cluster remains and is typically illustrated by a dendrogram (tree diagram). The dendrogram provides a visual summary of the clustering process, presenting a picture of the groups and their proximity, with a dramatic reduction in dimensionality of original data. The Euclidean distance usually provides the similarity between two samples and a distance can be represented by the difference between analytical values from samples. In the present study, hierarchical CA was adopted to the standardized data using Ward’s method, with Euclidean distance as a measure of similarity. The Ward method applies an analysis of variance approach to assess the distances between clusters to minimize the sum of squares of any two clusters that can be formed at each step. The spatial variability of water quality in the lake was determined from hierarchical CA using the linkage distance [17–19].

### 2.3. Principal Component Analysis/Factor Analysis

Principal component analysis is a data analysis method focused on a particular collection of variables. Consider the form of the first principal component. The score for individual i on component, *c_i_*_1_, uses weight *w*_11_, ….., *w_p_*_1_ in the linear combination:

(1) $$c_{i1}=y_{i1}w_{11}+y_{i2}w_{22}+\ldots +y_{\text{ip}}w_{p1}$$

The linear combination is chosen so that the sum of squares of *c*_1_ is as large as possible subject to the condition that *w*_11_^2^ + …..+ *w_p_*_1_^2^ = 1. The second principal component is another linear combination of *y_j_*:

(2) $$c_{i2}=y_{i1}w_{12}+y_{i2}w_{22}+\ldots +y_{\text{ip}}w_{p2}$$

where the variance *c*_2_ is the maximal, subject to the conditions that corr (*c*_1_, *c*_2_ )=0 and that *w*_12_^2^ + …...+ *w_p_*_2_^2^ = 1. The criterion of summarizing the information in *p* variables by a few components is valuable as a means of reducing the number of variables needed in an analysis [20].

FA follows PCA. FA focuses on reducing the contribution of less significant variables to simplify even more of the data structure coming from PCA. This purpose can be implemented by rotating the axis defined by PCA based on well established rules, and constructing new variables, also called varifacrors (VFs). PCA of the normalized variables was performed to extract significant PCs and to further reduce the contribution of variables with minor significance; these PCs were subjected to varimax rotation (raw) generating VFs [21,22].

The FA can be written as:

(3) $$y_{\text{ji}}=f_{j1}z_{i1}+f_{j2}z_{i2}+\ldots +f_{\text{jm}}z_{\text{im}}+e_{\text{ij}}$$

where *y* is the measured variable, *f* is the factor loading, *z* is the factor score, *e* is the residual term accounting for errors, *i* is the sample number and *m* is the total number of factors. The multivariate statistical technique calculations were implemented using STATISTICA 8 [23] and Microsoft Office Excel 2007.

### 2.4. Geostatistical Mapping

Geostatistical mapping can be defined as the analytical production of maps by using field observations, auxiliary information and a computer program that generates predictions. The isotropic semivariogram are estimated to characterize the relationship between general spatial dependence and distance among the observations. Different semivariogram models, e.g., exponential and Gaussian models, nested with nugget effects are selected separately with respect to different principle components or factor scores. The optimal parameters for semivariogram models are calculated by the weighted least squares method [24]. Despite the concerns about the spatial non-orthogonality, the cross-correlations between different principle components or factor scores are calculated [25,26]. It shows that the cross-correlations increase as the spatial lags increases; however, the maximum cross-correlations are still small and less than 0.4. This study then assumes the spatial orthogonality of the principle components as well as the factor scores. The use of simple kriging usually requires the knowledge of the underlying space/time trend of the attributes of concern. However, it is not available for the modeling of “transformed” variables in this study. In these cases, many studies use nonparametric method for the trend modeling. Therefore, in this study, ordinary kriging is used for the spatial mapping which considers a non-parametric trend as well the spatial association among the attributes concurrently. All the geostatistical analysis computations of this study were performed on SEKSGUI, which is freely and publicly available [27].

## 3. Results and Discussion

The measured results of 14 physico-chemical water quality parameters at eight sampling stations from August 2008 to June 2010 in the YYL are presented in Table 1.

### 3.1. Spatial Similarity with CA

**Figure 2.**
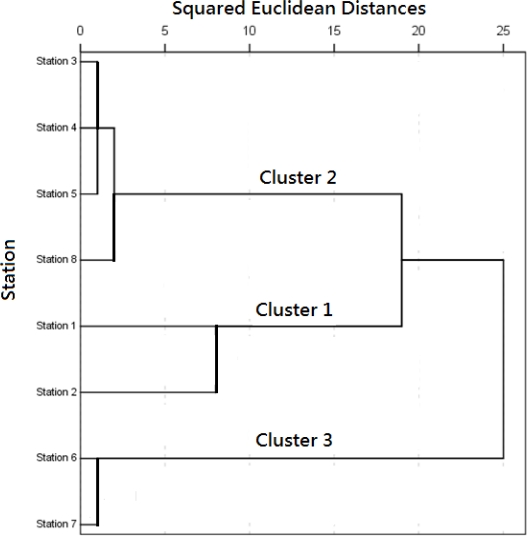
Dendrogram of cluster analysis for sampling stations accroding to water quality paramters of YYL.

Cluster analysis was applied to find out the similarity groups between the sampling stations. It produced a dendogram (Figure 2), grouping all eight sampling stations into three statistically meaningful clusters.

The two measurement stations (1 and 2) are regarded as the cluster 2 which comprises the shallow area. Stations 3, 4, 5, and 8 are cluster 1 which corresponds to the middle water depth. Stations 6 and 7 belonging to the deep zone which constitutes cluster 3. The results show that the CA technique is useful for classification of lake waters, hence, the number of sampling sites and respective cost can be diminished in future monitoring plans. There are other reports [28–30], with similar water quality program results.

### 3.2. Principal Component Analysis and Pollution Identification

Pattern recognition of correlations among 14 parameters was best summarized by PCA/FA. The Bartlett test was used on the data set to examine the suitability of these data for PCA/FA. In this study, the covariance matrix coincided with the correlation matrix which was presented in Table 2, because FA/PCA was applied to normalized data. Overall, the correlations between variables were relatively weak. There are some positive correlations between some variables such as TP, NH_4_-N, TN, TSS, Chl-a, and so on. The negative correlations were revealed between some variables such as DO, Temp, NH_4_-N, TN, Chl-a, Turb, and so on. Correlation coefficients of two elements were very useful, because they numerically represented the similarity between two elements of the two water quality variables. This also indicated that PCA could successfully reduce the dimensionality of the original data set. Therefore factor analysis of the present data set further reduced the contribution of less significant variables obtained from PCA.

**Figure 3.**
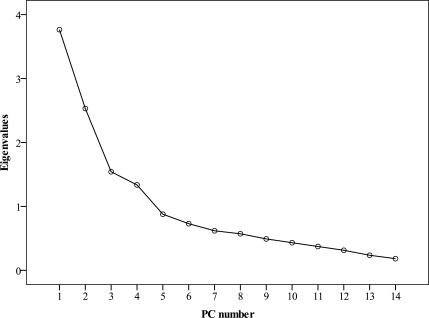
Scree plot of the characteristic roots (eigenvalues) of principal component analysis.

The Scree plot (shown in Figure 3) was applied to identify the number of PCs to be retained to understand the underlying data structure. Based on the Scree plot and the eigenvalues >1 criterion, four factors were chosen as principal factors, explaining 65.52% of the total variance in the data set. The corresponding VFs, variables loadings, eigenvalues, and explained variance are presented in Table 3.

Liu *et al.* [31] classified the factor loadings as “strong”, “moderate”, and “weak”, corresponding to absolute loading values of >0.75, 0.75–0.50, and 0.50–0.30, respectively. The first factor (VF1), explaining 26.89% of total variance, had moderate positive loadings on TP, NH_4_-N, TSS, Chl-a, and Turb (turbidity). Because the NH_4_-N concentration is a nutrient source for chlorophyll a growth, VF1 represented nitrogen source. VF2, which explained 18.08% of total variance, had a moderate positive loading on R (rainfall), WS (wind speed), TN, and pH and represents meteorological factors. VF3, explaining 11.02% of total variance, has a moderate positive loading on Ke, SD, and Turb (turbidity). This factor represents the contribution of turbidity effects in the water column. VF4, explaining 9.54% of total variance, had a moderate positive loading on NO_3_-N and water temperature and represented the nitrate factor. The analyzed results revealed that FA/PCA can serve as an important means to identify the main factors affecting water quality in the alpine lake.

### 3.3. Geostatistical Mapping

**Figure 4.**
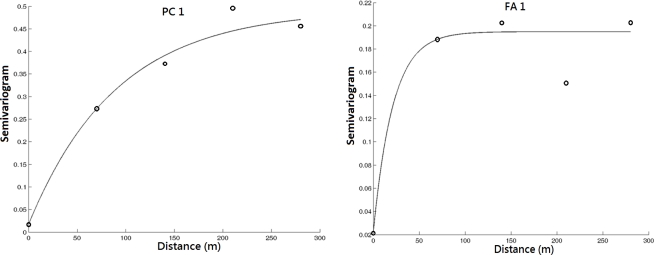
The experimental and modeled variograms of PC1 and FA1.

**Figure 5.**
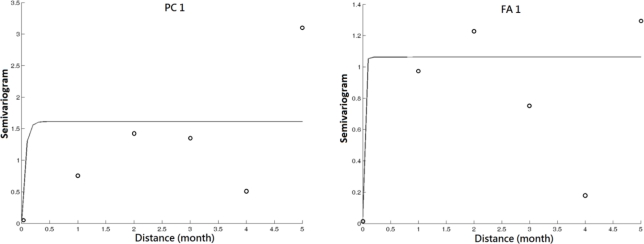
Variograms in time for PC1 and FA1.

**Figure 6.**
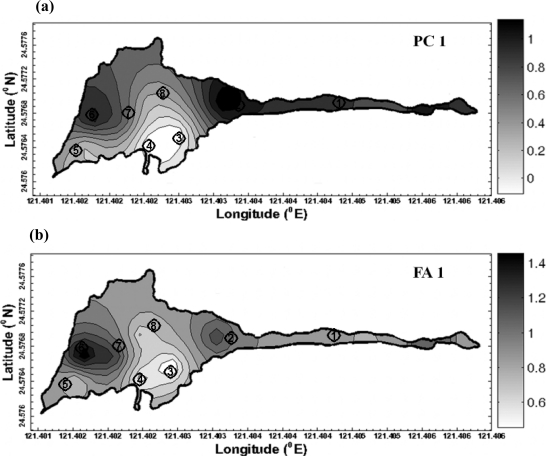
Spatial distribution of **(a)** first principle component and **(b)** first factor score at the time on the measured data of September 12, 2009.

Geostatisitcal techniques were used for the mapping of principle components and factor scores over the study area. Due to the long period between each observation campaign, the temporal correlation among the observations is assumed to be ignorable in this analysis. Table 4 shows that the spatial dependence structure varies across the identified contributing factors by the common multivariate analysis. It implies the variation of spatial patterns of impacts to water quality from the contributing factors. Among them, the impact of nitrogen nutrients changes more significantly over space than other contributing factors. The experimental and modeled variograms of PC1 and FA1 are shown in Figure 4. The variogram figure in time for PC1 and FA1 is also presented in Figure 5. It is clear that the variogram value approximates to sill in cases of the temporal lags in month among observations larger than 0. It implies the low correlation between the observations collected in different months. The contaminants from nitrogen nutrient are more localized as shown in Figure 6. On the other hand, the effects from the sunlight, organic matter, and nitrate nutrition present much smoother variations across the study area. This implies the sources of these contributors are more homogeneously distributed over the lake. It is noticeable that the range of the semivariogram model of second principle component is excessively larger than those of the models of other factors. It implies that the meteorological effects derived from PCA contribute a relatively large scale variation of water quality in space with respect to the scale of the study area.

The spatial distribution of the PC and FA can vary over time. Our analysis shows that the spatial distributions of PC (or FA) of the observations collected in the same month are generally similar. As for the PC obtained at different months, their spatial distribution can be distinct. This variability can result from meteorological condition and physico-chemical characteristics.

**Figure 7.**
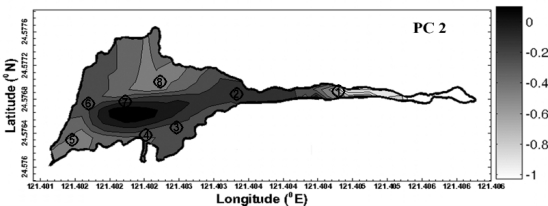
Spatial distribution of second principle component by ordinary kriging method on the measured data of February 14, 2009.

The general characteristics can be seen in Figure 7 in which a clear increasing trend from south to north of principle component is shown.

## 4. Conclusions

Water quality data collected from eight monitoring stations located around the subtropical alpine Yuan-Yang Lake in Taiwan have been examined by unsupervised pattern recognition (CA) and display methods (PCA/FA) to yield correlations between variables and water quality similarity in the lake. Cluster analysis confirmed the existence of three types of water quality (*i.e.*, shallow, middle and deep zones of the lake). The PCA and FA assisted to extract and recognize the factors or origins responsible for water quality variations. PCA/FA identified four latent factors that explained 65.52% of total variance, namely nitrogen source, meteorological factor, turbidity effect, and nitrate factor, respectively. Geostatisitcal techniques were used for the mapping of principle components and factor scores in the lake. The results revealed that the impact of nitrogen nutrients changes more significantly over space than other contributing factors. It means that nitrogen sources consist of important contribution to affect the water quality of the lake. Thus, this study illustrated the usefulness of multivariate statistical and geostatistical techniques for the analysis and interpretation of complex data set, water quality assessment, and identification of important contribution in nutrient source in the YYL.

## Figures and Tables

**Table 1.** Results of water quality parameters at eight sampling in the YYL.

**Parameter**	**Abbreviation**	**Station 1**	**Station 2**	**Station 3**	**Station 4**	**Station 5**	**Station 6**	**Station 7**	**Station 8**
Temperature (°C)	Temp	12.4 ± 2.88 .	13.63 ± 3.80	14.30 ± 3.41	14.41 ± 3.66	14.67 ± 3.62	13.86 ± 3.24	13.83 ± 3.49	14.47 ± 3.75
Dissolved Oxygen (mg/L)	DO	5.82 ± 0.89	6.49 ± 0.92	6.85 ± 0.75	6.79 ± 1.08	6.57 ± 1.26	6.11 ± 1.40	6.01 ± 1.30	6.78 ± 0.82
Secchi Depth (m)	SD	0.65 ± 0.12	0.86 ± 0.14	1.79 ± 0.39	1.69 ± 0.40	1.79 ± 0.44	1.95 ± 0.41	1.92 ± 0.39	1.84 ± 0.36
Total Phosphorus (mg/L)	TP	0.011 ±.005	0.014 ± 0.008	0.012 ± 0.006	0.011 ± 0.006	0.009 ± 0.004	0.009 ± 0.004	0.010 ± 0.004	0.009 ± 0.003
Total Nitrogen (mg/L)	TN	0.528 ± 0.169	0.544 ± 0.219	0.452 ± 0.196	0.427 ± 0.115	0.432 ± 0.144	0.454 ± 0.184	0.448 ± 0.166	0.422 ± 0.169
Ammonium Nitrogen (mg/L)	NH_4_-N	0.080 ± 0.112	0.078 ± 0.057	0.074 ± 0.039	0.051 ± 0.037	0.055 ± 0.031	0.097 ± 0.085	0.100 ± 0.102	0.077 ± 0.089
Nitrate Nitrogen (mg/L)	NO_3_-N	0.111 ± 0.053	0.071 ± 0.038	0.083 ± 0.045	0.092 ± 0.042	0.091 ± 0.044	0.097 ± 0.044	0.095 ± 0.041	0.097 ± 0.045
Total Suspended Solids (mg/L)	TSS	5.38 ±.02	5.87 ± 3.88	3.79 ± 2.73	3.19 ± 1.95	4.18 ± 2.84	3.44 ± 3.07	3.90 ± 3.73	3.57 ± 2.74
Turbidity (NTU)	Turb	14.10 ± 7.60	16.24 ± 7.31	15.18 ± 6.36	15.25 ± 6.95	16.14 ± 7.72	18.23 ± 7.81	18.52 ± 8.65	15.83 ± 6.45
Chlorophyll *a* (μg/L)	Chl-a	4.20 ± 3.44	7.33 ± 6.68	4.50 ± 3.17	3.49 ± 2.05	3.11 ± 1.98	6.39 ± 5.68	7.78 ± 10.14	3.83 ± 2.35
pH (pH unit)	pH	5.89 ± 0.43	6.30 ± 0.45	6.42 ± 0.39	6.43 ± 0.29	6.49 ± 0.38	6.41 ± 0.29	6.48 ± 0.32	6.48 ± 0.34
Light attenuation coefficient (m^−1^)	Ke	4.78 ± 2.52	4.87 ± 2.48	2.68 ± 1.17	2.58 ± 1.30	2.67 ± 1.27	2.84 ± 1.26	2.37 ± 0.87	4.35 ± 1.97
Wind Speed (m/s)	WS	0.744 ± 0.182	0.744 ± 0.182	0.744 ± 0.182	0.744 ± 0.182	0.744 ± 0.182	0.744 ± 0.182	0.744 ± 0.182	0.744 ± 0.182
Rainfall (mm)	R	4.318 ± 7.048	4.318 ± 7.048	4.318 ± 7.048	4.318 ± 7.048	4.318 ± 7.048	4.318 ± 7.048	4.318 ± 7.048	4.318 ± 7.048

Note: Values represent mean ± standard deviation.

**Table 2.** Correlation matrix of water quality parameters of YYL.

	**Temp**	**DO**	**WS**	**R**	**SD**	**TP**	**NH_4_-N**	**NO_3_-N**	**TN**	**TSS**	**Chl-a**	**Turb**	**pH**	**Ke**
**Temp**	1													
**DO**	−0.38 **	1												
**WS**	−0.07	−0.04	1											
**R**	0.1	0.1	−0.77 **	1										
**SD**	−0.12	0.26 *	−0.02	0.02	1									
**TP**	0.24 *	−0.15	−0.32 **	−0.26 *	−0.27 *	1								
**NH_4_-N**	0.30 **	−0.27 *	−0.25 *	−0.32 **	−0.18	0.37 **	1							
**NO_3_-N**	−0.26 *	−0.10	0.21	0.22 *	0.13	−0.28 **	0.04	1						
**TN**	0.26 *	−0.46 **	0.17	−0.21	−0.23 *	0.24 *	0.35 **	0.16	1					
**TSS**	0.15	−0.23 *	−0.33 **	−0.32 **	−0.18	0.51 **	0.37 **	0.12	0.25 *	1				
**Chl-a**	0.17	−0.34 **	−0.34 **	0.28 *	−0.14	0.39 **	0.48 **	−0.10	0.27 *	0.59 **	1			
**Turb**	0.36 **	−0.48 **	−0.18	−0.20	0.01	−0.14	0.55 **	0.16	0.36 **	0.29 **	0.42 **	1		
**pH**	0.02	0.36 **	−0.19	0.17	0.38 **	−0.05	−0.11	−0.30 **	−0.37 **	−0.18	−0.09	−0.18	1	
**Ke**	−0.11	−0.15	−0.01	0.14	−0.38 **	0.04	−0.08	0.07	0.24 *	0.11	0.13	−0.08	−0.36 **	1

* Values are statistically significant at p < 0.01;

** values are statistically significant at p < 0.05.

**Table 3.** Loading of 14 parameters on significant VFs for water quality data set.

**Parameters**	**Four significant PCs**			
	**VF1**	**VF2**	**VF3**	**VF4**
Temp	0.465	0.038	0.309	−0.623
DO	−0.582	0.437	−0.205	0.171
WS	−0.409	−0.696	0.201	−0.237
R	0.383	0.735	−0.105	0.218
SD	−0.367	0.330	0.581	0.254
TP	0.610	0.224	−0.309	−0.218
NH_4_-N	0.718	0.096	0.252	0.051
NO_3_-N	−0.043	−0.460	0.299	0.704
TN	0.536	−0.543	0.118	−0.105
TSS	0.698	0.111	−0.163	0.310
Chl-a	0.737	0.133	−0.047	0.148
Turb	0.655	−0.067	0.533	0.110
pH	−0.314	0.649	0.217	−0.214
Ke	0.162	−0.429	−0.627	0.132
Eigenvalue	3.76	2.53	1.54	1.34
Percentage of total variance	26.89	18.08	11.02	9.54
Cumulative percentage of variance	26.89	44.96	55.98	65.52

**Table 4.** Variogram models used for spatial mapping.

**Variables**	**Variogram models**
PC1	Nugget[0.031] + Exponential[0.466, 287.106]
PC2	Nugget[0.007] + Gaussian[5.004, 1116.3]
PC3	Nugget[0.036] + Gaussian[1.443, 259.137]
PC4	Nugget[0.018] + Gaussian[0.305, 215.136]
FA1	Nugget[0.038] + Exponential[0.157, 65.983]
FA2	Nugget[0.003] + Exponential[0.080, 185.810]
FA3	Nugget[0.010] + Gaussian[2.056, 383.165]
FA4	Nugget[0.010] + Gaussian[0.409, 288.627]

Note: The notations that Nugget[ *s*_1_ ] + Exponential(or Gaussian)[ *s*_2_, *r*_2_ ] denote the nest model of nugget model effect of sill *s*_1_ and exponential (or Gaussian) model of sill *s*_2_ and range *r*_2_ in meters. PC: Principal Component; FA: Factor Analysis.

## References

[1] Zeng X, Rasmussen TC (2005). Multivariate statistical characterization of water quality in Lake Lanier, Georgia, USA. J. Environ. Qual.

[2] Heikka RA (2007). Multivariate monitoring of water quality: A case study of Lake Simple, Finland. J. Chemomet.

[3] Nakasone H (2009). Effect on water quality in irrigation reservoir due to application reduction of nitrogen fertilizer. Paddy Water Environ.

[4] Palma P, Alvarenga P, Palma VL, Fernandes RM, Soares AMVM, Barbosa IR (2010). Assessment of anthropogenic sources of water pollution using multivariate statistical techniques: A case study of Alqueva’s reservoir, Portugal. Environ. Monit. Assess.

[5] Singh KP, Malik A, Mohan D, Sinha S (2004). Multivariate statistical techniques for the evaluation of spatial and temporal variations in water quality of Gomti river (India): A case study. Water Res.

[6] Li R, Dong M, Zhao Y, Zhang L, Cui Q, He W (2007). Assessment of water quality and identification of pollution sources of plateau lakes in Yunnan (China). J. Environ. Qual.

[7] Kazi TG, Arain MB, Jamali MK, Jalbani N, Afridi HI, Sarfraz RA, Baig JA, Shah AQ (2009). Assessment of water quality of polluted lake using multivariate statistical techniques: A case study. Ecotox. Environ. Safe.

[8] Singh KP, Malik A, Sinha S (2005). Water quality assessment and apportionment of pollution sources of Gomti river (India) using multivariate statistical techniques: A case study. Anal. Chim. Acta.

[9] Kim JH, Choi CM, Kim SB, Kwun SK (2009). Water quality monitoring and multivariate statistical analysis for rural streams in South Korea. Paddy Water Environ.

[10] Chehata M, Jasinski D, Monteith MC, Samuels WB (2007). Mapping three-dimensional water-quality data in the Chesapeake Bay using geostatistics. J. Am. Water Resour. Assoc.

[11] Todd MJ, Lowrance RR, Goovaerts P, Vellidis G, Pringle CM (2010). Geostatistical modeling of the spatial distribution of sediment oxygen demand within a Coastal Plain backwater watershed. Geoderma.

[12] Lopez-Granados F, Jurado-Exposito M, Pena-Barragan JM, Garcia-Torres L (2005). Using geostatistical and remote sensing approaches for mapping soil properties. Eur. J. Agron.

[13] Nour MH, Smit DW, El-Din MG (2006). Geostatistical mapping of precipitation: Implication for rain gauge network design. Water Sci. Technol.

[14] Sauquet E (2006). Mapping mean annual river discharge: Geostatistical development for incorporating river network dependencies. J. Hydrol.

[15] Wackernagel H, Lajaunie C, Blond N, Vautard R (2004). Geostatistical risk mapping with chemical transport model output and ozone station data. Ecol. Model.

[16] USEPA (1983). Methods for Chemical Analysis of Water and Waste.

[17] Wunderlin DA, Diaz MP, Ame MV, Pesce SF, Hued AC, Bistoni MA (2001). Pattern recognition techniques for the evaluation of spatial and temporal variations on water quality. A case study: Suquira river basin (Cordoba-Argentina). Water Res.

[18] Simeonov V, Stratis JA, Samara C, Zachariadis G, Vousta D, Anthemidis A, Sofoniou M, Kouimtzis Th (2003). Assessment of the surface water quality in Northern Greece. Water Res.

[19] Kowalkowski T, Zbytniewski R, Szpejna J, Buszewski B (2006). Application chemometrics in river water classification. Water Res.

[20] Helena B, Pardo R, Vega M, Barrado E, Fernandez JM, Fernandez L (2000). Temporal evolution of groundwater composition in an alluvial aquifer (Pisuerga river, Spain) by principal component analysis. Water Res.

[21] Brumelis G, Lapina L, Nikodemus O, Tabors G (2000). Use of an artificial model of monitoring data to aid interpretation of principal component analysis. Environ. Modell. Softw.

[22] Abdul-Wahab SA, Bakheit CS, Al-Alawi SM (2005). Principal component and multiple regression analysis in modelling of ground-level ozone and factors affecting its concentration. Environ. Modell. Softw.

[23] (2007). STATISTICA (data analysis software system), Version 8.

[24] Cressie N (1985). Fitting variogram models by weighted least-squares. J. Int. Assoc. Math. Geol.

[25] Goovaerts P (1977). Geostatistics for Natural Resources Evaluation.

[26] Wackernagel H (2003). Multivariate Geostatistics: An Introduction with Applications.

[27] Yu HL, Kolovos A, Christakos G, Chen JC, Warmerdam S, Dev B (2007). Interactive spatiotemporal modelling of health systems: The SEKS–GUI framework. Stoch. Environ. Res. Risk Assess.

[28] Kim JH, Kim RH, Lee J, Cheong TJ, Yum BW, Chang HW (2005). Multivariate statistical analysis to identify major factors governing groundwater quality in the coastal area of Kimje, South Korea. Hydrol. Process.

[29] Shrestha S, Kazama F (2007). Assessment of surface water quality using multivariate statistical techniques: A case study of the Fuji river basin, Japan. Environ. Modell. Softw.

[30] Zaharescu DG, Hooda PS, Soler AP, Fernandez J, Burghelea CI (2009). Trace metals and their source in the catchment of the high altitude Lake Respomuso, Central Pyrenees. Sci. Total Environ.

[31] Liu CW, Lin KH, Kuo YM (2003). Application of factor analysis in the assessment of groundwater quality in a Blackfoot disease area in Taiwan. Sci. Total Environ.

